# Chalk Yeasts Cause Gluten-Free Bread Spoilage

**DOI:** 10.3390/microorganisms13061385

**Published:** 2025-06-14

**Authors:** Michela Pellegrini, Lucilla Iacumin, Francesca Coppola, Federica Barbieri, Chiara Montanari, Fausto Gardini, Giuseppe Comi

**Affiliations:** 1 Department of Agricultural, Food, Environmental and Animal Science, University of Udine, 33100 Udine, Italy; michela.pellegrini@uniud.it (M.P.); lucilla.iacumin@uniud.it (L.I.); 2 Food Sciences Institute, National Research Council, Via Roma, 64, 83100 Avellino, Italy; fracop93@gmail.com; 3 Department of Agricultural and Food Sciences, University of Bologna, 47521 Cesena, Italy; federica.barbieri16@unibo.it (F.B.); chiara.montanari8@unibo.it (C.M.); fausto.gardini@unibo.it (F.G.)

**Keywords:** chalk yeasts, gluten-free bread, spoilage, preservatives, volatile compounds

## Abstract

Four different yeast strains were isolated from industrial gluten-free bread (GFB) purchased from a local supermarket. These strains, including *Hyphopichia burtonii*, *Wickerhamomyces anomalus*, *Saccharomycopsis fibuligera*, and *Cyberlindnera fabianii*, are responsible for spoilage, which consists of white powdery and filamentous colonies due to the fragmentation of hyphae into short-length fragments (dust-type spots) that is typical of the spoilage produced by chalk yeasts. The isolated strains were identified using genomic analysis. Among them, *C. fabianii* was also isolated, which is a rare ascomycetous opportunistic yeast species with low virulence attributes, uncommonly implicated in bread spoilage. The yeast growth was studied in vitro on Malt Extract Agar (MEA) at two temperatures (20 and 25 °C) and at different Aws (from 0.99 to 0.90). It was inferred that the temperature did not influence the growth. On the contrary, different Aws reduced the growth, but all the yeast strains could grow until a minimum Aw of about 0.90. Different preservatives (ethanol, hop extract, and sorbic and propionic acids) were used to prevent the growth. In MEA, the growth was reduced but not inhibited. In addition, the vapor-phase antimicrobial activity of different preservatives such as ethanol and hop extract was studied in MEA. Both preservatives completely inhibited the yeast growth either at 20 or at 25 °C. Both preservatives were found in GFB slices. Contrary to hop extract, 2% (*v*/*w*) ethanol completely inhibited all the strains. The spoilage was also confirmed by the presence of various compounds typically present in yeasts, derived from sugar fermentation and amino acid degradation. These compounds included alcohols, ketones, organic acids, and esters, and they were identified at higher concentrations in the spoiled samples than in the unspoiled samples. The concentration of acetic acid was low only in the spoiled samples, as this compound was consumed by yeasts, which are predominately present in the spoiled samples, to produce acetate esters.

## 1. Introduction

The increased prevalence of celiac disease, as well as the increased proportion of the population turning to healthier diets, has led to a growing demand for gluten-free products (GFPs) in the market, especially bakery products [1,2,3,4]. Due to dietary restrictions, people with celiac disease constitute a special group of consumers with specific attitudes and needs, and they are driving the gluten-free bread (GFB) industry [5,6,7]. Bread represents one of the main GFPs. For this reason, in recent years, the main focus of the research on increasing GFB quality has been the improvement in the technological characteristics of gluten-free flours and GFB production. In particular, the sensorial quality of gluten-free bread and other bakery goods like cookies, muffins, or sponge cakes has reached high levels and has satisfied celiac consumers. This is also demonstrated by the various papers about GFB production and technology [8,9,10]. However, little or no information can be found about the impact of spoilage of GFB on celiac consumers. Usually the spoilage of gluten bread (GB) is a major concern in the food industry and leads to large food losses [11]. The major losses occur for industrial GB, e.g., toast bread and modified-atmosphere-packaged (par-baked) bread, which is stored for a longer period than traditional fresh bread [11,12]. Yeasts and molds are ubiquitous microorganisms that represent the main cause of spoilage of bread around the world [12,13,14,15]. The spoilage is caused by the fungal species of the genera of *Penicillium* or *Aspergillus* [16] and by chalk yeasts, also called chalk molds; although they are yeasts [17], they are characterized by the ability to produce white powdery and filamentous colonies due to the fragmentation of their hyphae into short-length fragments (dust-type spots) [16,18,19,20,21], resembling a lot like molds. In particular, chalk yeasts are most common on sliced and rye bread. Yeasts and molds easily grow on bread due to its high moisture and richness of nutrients [12,14,17,19], resulting in the spoilage of bread that consists of several defects, including visible molding, off-flavors, and odors, thus causing significant economic losses within the bakery industry [12,13]. Among chalk yeasts, the dominant species include *S. fibuligera* [20,21], *H. burtonii* [17,20,22], *Zygosaccharomyces bailli*, and *Saccharomyces cerevisiae* [13]. In addition, *W. anomalus* (formerly known as *Pichia anomala*) is also responsible for the spoilage of bread, but it is not a chalk yeast, even though some authors consider it as a part of the chalk yeast group [18,23]. Recently, Colautti et al. [24] isolated *C. fabianii*, an uncommon yeast, which was responsible for GB spoilage. It produced different compounds, including alcohols, organic acids, and esters, and a white powder similar to the spoilage produced by chalk yeasts. The type of packaging and the addition of ethanol as a preservative do not stop the chalk yeast growth. *W. anomalus*, *S. fibuligera*, and *H. burtonii* were found to spoil modified-atmosphere-packaged par-baked bread [17], and they were also frequently isolated from spoiled industrial bread packaged in the air [19]. *W. anomalus* is majorly responsible for ethyl acetate production, which confers an undesirable smell to bread, identified as “chemical odor”, and results in consumer complaints [13,18]. The origin of bread contamination is not well known, but surely it occurs after baking, as the cooking temperature, reaching over 180 °C, can kill both molds and yeasts. According to Giannone et al. [19], fungal cells are carried by bioaerosols within the bakery plant. Then, they grow on the bread during storage either at room or at refrigeration temperature regardless of the type of packaging (air, under vacuum, or MAP) or preservative added. In Italy, according to the Decreto Ministeriale [25], to increase the shelf-life of industrial bread packaged in air or in MAP, aliquots of ethanol can be added, with a maximum level of 2% with respect to the dry substance. Moreover, ethanol at this concentration does not stop the chalk yeast growth. Again, chalk yeasts can have antifungal properties against spoilage molds. Coda et al. [26] demonstrated that using dough fermented with *W. anomalus* LCF1695 could extend the mold-free shelf-life of bread slices to up to 28 days at room temperature; consequently, Aloui et al. [27] proposed that this species is a potential biocontrol agent for *Penicillium* species. To prevent the spoilage of bread by yeasts, it is important to fully comprehend the influence of environmental conditions on their growth. Finally, a great number of papers about GB spoilage are present in the scientific literature, while no literature data are available on the growth of chalk yeasts and the influence of environmental growth conditions on GFB. Therefore, the aims of this study were to assess the spoilage of GFB and to contribute to the existing scientific knowledge on GFB spoilage by chalk yeasts.

## 2. Material and Methods

### 2.1. Microbial Analysis

Three different lots of spoiled GFB, made with the same recipe consisting of maize starch, water, sourdough, maize flour, rice flour, psyllium seed husk (vegetable fiber), thickener, dextrose, modified cellulose, soy protein, sunflower oil, extra virgin olive oil, yeast extract, tartaric acid, citric acid, and salt, were collected from a local high-quality supermarket and analyzed. The bread was stored at room temperature, as indicated on the label. Each lot included 10 bread packages of 500 g each. Lots 1 and 2 included 7 out of 10 and Lot 3 included 8 out of 10 spoiled bread packages. From each lot, 5 spoiled packages were collected and analyzed. All the products were packaged in polypropylene bags in modified atmosphere packaging consisting of 100% CO_2_. The surface of each spoiled bread sample presented spots (3–4 spots of each bread sample) consisting of white powdery or filamentous colonies. A part of the spots was directly streaked on Malt Extract Agar (MEA, Oxoid, Milan, Italy) and incubated at 25 °C for up to 5 days. From each lot, 10 spoiled bread samples collected from spoiled packages were also analyzed using decimal dilution.

Each bread sample was mixed in a stomacher bag, and 25 g of each sample was serially diluted with saline–peptone water (PW, 8 g/L NaCl, 1 g/L bacteriological peptone, Oxoid, Milan, Italy, 1000 mL distilled water) in a stomacher bag apparatus. An aliquot of 1 mL of each serial dilution was plated in MEA as a single layer and incubated at 25 °C for up to 5 days.

From each MEA plate, 5 different morphological yeast groups were isolated, purified, and stored on MEA slants. Consequently, a total of approximately 150 strains were collected and then identified.

### 2.2. Identification of Isolated Yeasts

One milliliter of an overnight culture of each isolate was centrifuged at 14,000× *g* for 10 min at 4 °C to pellet the cells, and the pellet was subjected to DNA extraction according to Iacumin et al. [28], with the addition of only lysozyme (50 mg/mL, Sigma, Milan, Italy) for cell lysis. Yeasts were identified using molecular methods such as Nested PCR (2-step amplification) and DGGE analysis. For the first PCR process, primers NL1, 50-GCC ATA TCA ATA AGC GGA GGA AAA G-30 and NL4, 50-GGT CCG TGT TTC AAG ACG G-30 [28] were employed. The reaction mix was as follows: 10 mM Tris-HCl (pH 8.3), 50 mM KCl, 1.5 mM MgCl_2_, 0.2 mM each dNTP, 1.25 U of Taq Polymerase (Applied Biosystem, Milan, Italy), and 0.2 mM of each primer. A total of 100 ng of DNA was added to the reaction mixture. Reactions were run for 30 cycles: denaturation was performed at 95 °C for 60 s, annealing at 48 °C for 45 s, and extension at 72 °C for 60 s. An initial denaturation at 95 °C for 5 min and a final extension for 7 min at 72 °C were used. One microliter of the first step of PCR was subjected to a second set of 35 cycles of PCR using a pair of primers (NL1 and LS2, 50-ATT CCC AAA CAA CTC GAC TC-30) [29] that amplified a 240 bp sequence within the original amplicon. A GC clamp (50-CGC CCG CCG CGC CCC GCG CCC GTC CCG CCG CCC CCG CCC G-30) was added to the forward primer (NL1) according to Sheffield et al. [30]. This reaction was performed in a final volume of 25 µL containing 10 mM Tris-HCl (pH 8.3), 50 mM KCl, 2 mM MgCl_2_, 0.2 mM each dNTP, 1.25 U of Taq Polymerase (Applied Biosystem, Milan, Italy), and 0.2 mM of each primer. The amplification conditions were as follows: 5 min at 95 °C, followed by 35 cycles of 1 min at 95 °C, 1 min at 52 °C, and 1 min at 72 °C. A final extension at 72 °C for 7 min was performed. The Dcode universal mutation detection system (Bio-Rad, Hercules, CA, USA), with a 0.8 mm thick polyacrylamide gel (8% [*w*/*v*] acrylamide–bisacrylamide [37.5:1]), was used for the DGGE analysis of amplicons. Electrophoresis was performed with a denaturant gradient from 30% to 60% (100% corresponds to 7 M urea and 40% [*w*/*v*] formamide), increasing in the direction of the electrophoretic run (120 V, 60 °C, 4 h). Gels were stained for 20 min in 1.25 x Tris–acetate–EDTA containing 1 x SYBR Green (Molecular Probes, Eugene, OR, USA). The pictures of the gels were digitally captured by the GeneGenius BioImaging System (SynGene). A reference pattern was designed consisting of 26S rRNA amplicons from 5 different yeast type strains: Saccharomyces bayanus (DSM 3774), Saccharomyces pastorianus (DSM 6581), Saccharomyces cerevisiae (ATCC 36024), Pichia membranifaciens (UCD 22), and Candida vini (UCD 36). With the inclusion of this reference pattern three times on each DGGE gel, the resulting band profiles could be digitally normalized by comparison with a standard reference, using the Gel Compare, version 4.1, software package (Applied Maths, Kortrijk, Belgium). Strains with the same DGGE profiles were grouped. For clustering, a UPGMA dendrogram was constructed, and similarities were expressed using the Pearson product–moment correlation coefficient (Gel Compare, version 4.1, software package, Applied Maths, Kortrijk, Belgium). Three representatives of each group were amplified with the primers NL1 and NL4, as described previously. After purification, products were sent to a commercial facility for sequencing (Eurofins MWG GmbH, Martinsried, Germany). Sequences were aligned with those in GeneBank with the Blast program [31] to determine the closest known relatives based on the partial 26S rRNA gene homology.

### 2.3. Inoculum Preparation

After identification, the yeast strains were stored at −80 °C in Malt Extract Broth (ME broth, Oxoid, Milan, Italy). The yeast cells were harvested by transferring a single loopful of cells into a 1.5 mL microcentrifuge tube containing a 0.85% (*w*/*v*) normal saline solution that was previously autoclaved at 121 °C for 15 min. Five strains of each yeast species, which were randomly selected, were grown separately in ME broth incubated at 25 °C for 72 h. Then, 0.5 mL of each ME culture was reinoculated in fresh ME broth and incubated at 25 °C for 72 h. One ml of each culture was diluted using the PW solution, and after mixing, the optical density (OD) of each yeast suspension was measured using a visible spectrophotometer (Unico, UV 2100, Shanghai, China) at 600 nm. A normal saline solution was used as a blank. The OD of each yeast suspension was adjusted to 0.1 at 600 nm. The concentration of each suspension was evaluated by plating 0.1 mL of each suspension on Malt Extract Agar (MEA, Oxoid, Milan, Italy), and incubating it at 25 °C for 72 h. Each suspension contained about 10^7^ CFU/mL. Then, 5 mL of the 5 suspensions of each chalk yeast species was mixed to form a cocktail and diluted to obtain a final concentration of approximately 10^7^ CFU/mL (OD 0.1 at 600 nm). These yeast suspensions represented the yeast cocktails used in all the experiments.

### 2.4. Growth of Chalk Yeasts (Colony Diameter Measured) on MEA

The growth was valued on MEA (Malt Extract Broth added with 1.5% agar, Oxoid, Milan, Italy). The pH value was adjusted to 5.7 units. The Aw was modified using different concentrations of NaCl, and the effect of this modification was assessed in the range of 0.90–0.99 with a 0.02 increment (the classical Aw value of commercially available bread) and at two temperatures (20 and 25 °C). Water activity (Aw) was determined using an Aqualab Hygromer AWVC (Rotronic, Milan, Italy) with an accuracy of ±0.003. Media were autoclaved for 15 min at 121 °C; then, 15 mL of MEA poured into the Petri dishes for inoculation. MEA was inoculated with 20 μL of the purified yeast suspensions containing 10^2^ colonies/20 μL that were spotted in the middle of each Petri dish [11]. The plates were incubated at 20 °C or 25 °C for 30 days, and colony diameters were measured using a digital caliper (Taurus Impact, Surville, France). Each combination of Aw and chalk yeasts was assessed in triplicate.

### 2.5. Antimicrobial Activity Versus Chalk Yeasts

The assay was performed using the method described by Virgili et al. [32] and Bleve et al. [33] and modified as follows: the experiments were performed on ME broth adjusted to pH 6.0. A top agar was prepared by mixing 10 mL of ME broth with 0.7% agar (MEA0.7), and different solutions (at the maximum level permitted by Italian Decreto Ministeriale [25]) of hop extract (2% *w*/*v*), sorbic acid (2000 ppm, Sigma Aldrich, Milan, Italy), propionic acid (2000 ppm, Sigma Aldrich, Milan, Italy), and ethanol (2% *v*/*w*, Sigma Aldrich, Milan, Italy) were added to obtain a thick, continuous layer on the plate surface. Hop extract solution, expressed as equivalents of chlorogenic acid (CAE) in mg per gram dry sample (final dilution: 4 mg of CAE/mL ethanol), was obtained according to Comi et al. [34]. After adding the antimicrobial solution, MEA0.7 was distributed into Petri plates containing 15 mL of MEA. Three 10 µL portions of *S. fibuligera*, *H. burtonii*, *W. anomalus*, and *C. fabianii* corresponding to 10^5^ and 10^4^ CFU/mL were then spotted onto each plate and incubated at 20 °C. Plates with MEA0.7 without antimicrobials, and inoculated with the chalky yeasts, were included as controls. Chalky yeast growth was expressed as the average measurement (cm) of two orthogonal diameters per colony after 30 days of incubation at 20 °C. The results were expressed as the time to detect the visibility in days (1 cm diameter) at 20 °C of the chalk yeast cocktails inoculated in MEA and stored for 1 month. Three replicates of each experiment were performed for each yeast.

### 2.6. Vapor-Phase Antimicrobial Activity of Different Compounds ([35], Modified)

In addition, the antimicrobial effect of ethanol and hop extract, using a vapor phase, was tested by modifying the method suggested by Petchwattana et al. [35]. The yeast suspensions were prepared as described in Section 2.3. The yeast cells were harvested by transferring a single loopful of cells into a 1.5 mL microcentrifuge tube containing 0.85% (*w*/*v*) normal saline solution that was previously autoclaved at 121 °C for 15 min. The optical density (OD) of the yeast cells was measured using a visible spectrophotometer (Unico, UV 2100, Shanghai, China) at 600 nm. A normal saline solution was used as a blank. The OD of each bacterial strain was adjusted to 0.1 at 600 nm. After decimal dilutions, an aliquot of 100 μL of 10^3^ CFU/mL dilution of each yeast species was transferred onto MEA. The culture dilution was spread on the plate using a spatula. This plate was employed to test the antimicrobial properties of different compounds (ethanol, hop extract). All the antimicrobial compounds were used at the following concentrations: 2, 4, and 6% (*v*/*w*), with 0.08, 0.16, and 0.48 mg/g MEA, respectively, and were attached to the inner side of the plate lid using a disk of Whatman paper and was allowed to release the vapor phase inside the plate. All the plates were sealed at the rim with Parafilm tape before incubating them at 20 and 25 °C for 3 days. The control contained MEA with the yeast culture without the compounds. The phase antimicrobial test was performed in triplicate.

### 2.7. Antimicrobial Activity of Hop Extract and Ethanol in Gluten-Free Bread

The yeast cocktails, as in Section 2.3, were spotted onto thirty-six gluten-free bread of about 50 g (9 bread pieces per yeast cocktail) and put into polypropylene bags. The bags were divided into two groups (Table 1). The hop extract solution (2% *v*/*w*, 0.08 mg/g product) was added to the first group, and the ethanol solution (2% *v*/*w*) was added to the second group. The polypropylene bags were packaged in 100% CO_2_ (MAP) using Cryovac packaging equipment (Duncan, IL, USA). A group of inoculated bread was also packaged without any antimicrobial solution as a control. The bags were stored at 20 °C, the simulated room temperature, and the yeast cocktail growth was checked every 2 days till 180 days.

### 2.8. Physicochemical Determination

The pH of the bread was measured directly by inserting a pH meter probe (Radiometer, København, Denmark) into the sample. The water activity (Aw) was determined using a Hygromer AWVC (Rotronic, Milan, Italy). The final values of all the above physicochemical parameters were expressed as the respective average measurements of six samples. The chemical parameters were determined according to A.O.A.C. [36]. The total fiber was determined by subtracting the sum of proteins, salts, carbohydrates, moisture, and fats from 100 g, expressed as g/100 g [37].

### 2.9. Volatile Compound Analysis

The profile in volatile compounds was evaluated both in the headspace of the packaging and in bread samples using a gas chromatographic–mass spectrometric technique coupled with solid-phase microextraction (GC-MS-SPME). First, the whole package was conditioned at 25 °C for 6 h, and then a fused silica fiber covered with 50/30 mm divinylbenzene/carboxen/polydimethylsiloxane (DVB/CAR/PDMS Stableflex, Supelco, Steiheim, Germany) was introduced into the headspace for 40 min at the same temperature. For the analysis of gluten-free bread, 3 g of samples were transferred in 10 mL sterilized vials, sealed with PTFE/silicon septa, and conditioned at 45 °C for 10 min and then the fiber was inserted in the vials to let absorb the volatile compounds for 40 min at the same temperature. In both cases, the fiber was then injected in the gas chromatogram for 10 min to desorb the compounds. The analysis was performed according to the protocol reported by Bancalari et al. [38] using a gas chromatography mass spectrometer coupled with the solid phase microextraction technique (SPME-GC-MS). An Agilent Hewlett-Packard 7890 GC gas chromatograph, equipped with an MS detector 5975C MSD (Hewlett-Packard, Geneva, Switzerland) and a CP-WAX 52CB 50 m, 0.32 mm, 1.20 µm fused silica capillary column was used (Agilent Technologies, Santa Clara, CA, USA). The chromatographic conditions were the following: injection temperature 250 °C; detector temperature, 250 °C; helium as carrier gas with a flow rate of 1 mL/min. The oven temperature program was as follows: 50 °C for 1 min, from 50 °C to 65 °C at 4.5 °C/min, from 65 °C to 230 °C at 10 °C/min, and then holding for 25 min. The identification of the VOC peaks was achieved using the Agilent Hewlett–Packard NIST 2011 mass spectral library (Gaithersburg, MD, USA) [39] and the data were expressed as a percentage of each molecule peak area on the total area of all the molecules identified. The results are the mean of three determinations for each sample.

### 2.10. Sensory Analysis

Sensorial analyses were performed by 20 non-professional and non-trained assessors (10 women and 10 men, representing food technology students aged between 22 and 24 years of age). The choice of non-professional tasters was mandatory because they represented typical consumers. Sensory analysis was performed based on the triangle test [40]. Three lots of unspoiled GFB, each represented by 10 samples, were evaluated by tasters who were asked to evaluate the influence of the presence of 2% ethanol in GFB on product quality. In short, three samples, two of which were identical, coded with three-digit numbers, were given in a randomized order, and the assessors were asked to identify the different samples. The test samples comprised packaged GFB with or without 2% ethanol. The samples were presented, wrapped in aluminum foil, to the tasters in a quiet room, and their responses were collected on a paper card.

The assessors were forced to give an answer and who identified the different samples were asked to indicate which sample they preferred and/or if both samples were acceptable. The statistical evaluation of the results was carried out according to ISO [40] and Stone and Sidel [41].

### 2.11. Statistical Analysis

Data were analyzed using the software Statistica 7.0 version 8 (Statsoft Inc., Tulsa, OK, USA; 2008). The values of the different parameters were compared using a one-way analysis of variance, and the means were then compared using Tukey’s honest significance test. Differences were considered significant at *p* < 0.05. Each physicochemical and microbial analysis included 10 samples either from spoiled or unspoiled goose sausages. Three samples were tested for volatilome analysis.

## 3. Results and Discussion

### 3.1. Physicochemical and Nutritional Aspects of GFB

Table 2 shows the physicochemical and nutritional characteristics of the commercial GFB. The obtained data confirm the traditional nutritional composition as determined in the studies on GFB reported by different authors [42], derived from product package labels and/or chemical analyses, or collected by searches conducted in grocery stores or on websites dealing with GF products [42]. However, GFB has different compositions depending on the regulations of each country, and it seems that some nutrients are not always declared. This study indicates a broad range of energy values and nutrient content, which resulted from the formulation and moisture content adopted by the producer. As shown, the moisture content is about 45%, and it depends on the level of water incorporated and retained after baking. In particular, it depends on the recipe, which could include maize starch, maize flour, vegetable fiber, cellulose, and soy protein, as demonstrated by references [42,43,44]. The levels of carbohydrate, protein, fat, and fiber content were 40, 4.6, 3, and 6.2%, respectively. The sugar and dietary fiber concentration may reflect the difference in the preference of consumers that can be country-specific or indicate a market development observed as the emergence of a wide range of GFB products with varying raw material compositions [42]. Comparing these data with those of other commercial GFBs, an extensive variability in the carbohydrate content can be observed. Carbohydrate levels represent the principal component of GFB due to the use of flours and starches as the main ingredients, and their concentration is similar either in white or in wholewheat bread, which contain 49.2% and 43.1% of carbohydrates, respectively [45,46]. As previously shown, the investigated GFB exhibited either sugar or fat values less than those of traditional commercial GFB. Although the GFB recipe contains sugar (dextrose), in the investigated GFB, its level was 2%; thus, the sugar level is less than that in other GFBs, in which it can vary from 0% to 24%. It can be assumed that the added sugars are consumed during the fermentation process [43]. The fat content of GFB, reaching 3%, is similar to that of white wheat bread and differs significantly from that of the commercial GFB, where it can range up to 19% [46]. The use of higher levels of fats is a common practice to improve the softness and consumer acceptance of bread [43,44]. However, the saturated fat content in GFB was low (0–5%) because of the use of vegetable oils instead of fats [43], as observed in Table 2. Indeed, the recipe of the investigated GFB included sunflower oil and extra virgin olive oil. The protein level is an important parameter in GFB production, representing 4.6% of the total composition. Usually, the protein concentration in commercial GFB is between 0 and 11%, which is lower than that of white wheat bread (8.8 g) [45].

The salt content of GFB is 1.1%; consequently, its sodium content is 650 mg/100 g, which is higher than the average value of 427 mg/100 g observed for white (490 mg/100 g) and wholewheat bread (450 mg/100 g) [45,46]. It is not necessary to use high amounts of salt and sodium in GFB, as the role that salt plays in traditional breadmaking, i.e., strengthening the dough, improving its handling, and reducing the yeast fermentation rate, is not necessary in GF breadmaking [42,43]. The salt effects are replaced by micronutrients present in the refined raw materials used to produce and to enrich commercially available GFB [43,44,47,48,49,50,51].

The fiber content of our GFB is 6.2%, which is similar to that of other commercially available GFBs, which vary widely in fiber content from 0% to 17% and depend on the variations in each formulation [43].

Finally, the high nutritional value of the investigated GFB is useful for human nutrition as well as for microorganisms. Indeed, three different lots of GFB were spoiled by yeast during their storage at room temperature.

### 3.2. Species Isolated in GFB

A total of four species were isolated from spoiled GFB (Table 3). The identification results showed a dominance of *S. fibuligera*, which is commonly found as the cause of chalky bread [52,53]. The second most isolated yeast was *C. fabianii* (formerly *P. fabianii*)*,* which was recently isolated from gluten bread (GB) by Colautti et al. [24]. This species is quite similar to *W. anomalus*. Indeed, Colautti et al. [24] initially identified *C. fabianii* as *W. anomalus* using traditional methods; however, through genomic analysis, it was accurately identified as *C. fabianii*, a rare ascomycetous opportunistic yeast species with low virulence attributes, uncommonly implicated in bread spoilage.

Then, the third most isolated species was *W. anomalus*, (formerly known as *P. anomala*). This species was the third most frequently reported foodborne yeast after *Saccharomyces cerevisiae* and *Debaryomyces hansenii* [54]. Finally, 15 isolated species were identified as *H. burtonii*. All the isolated species were chalk yeasts [18], except *C. fabianii*, and they were described in many scientific papers as common spoilers of European bread [52]. *H. burtonii* is described to be more common in British bread, whereas *S. fibuligera* are predominately present in French baguette. *W. anomalus* and *C. fabianii* can grow on different bread types [17].

All the isolated strains produced white pseudomycelia- and hypha-like structures and were responsible for the spread of white and powdery colonies that looked like sprinkled chalk dust on the surface of the product. In particular, the chalk-like appearance of *S. fibuligera* and *H. burtonii* is due to their typical growth structures resembling hyphae and/or pseudomycelia consisting of chains of budding yeast cells that do not separate after duplication [17]. Consequently, yeasts that produce white powdery and filamentous colonies due to the fragmentation of hyphae into short-length fragments (dust-type spots) are known as chalk yeasts. Furthermore, *C. fabianii* must be considered as a chalk yeast, as it is morphologically and structurally similar to *W. anomalus* and produces white hyphae and dust-type spots. This is the first report where *C. fabianii* was isolated from GFB.

The microbial analysis demonstrated that each strain was present in all the GFB samples. At 20 days of storage at room temperature (20–22 °C), the spoilage of GFB presented as small chalk spots that spread out across the whole product. The use of anaerobic conditions did not result in a long incubation period of the yeasts. Previous data mentioned that *H. burtonii* and *S. fibuligera* were also identified in GB packaged under a 100% CO_2_ atmosphere [17,20,21]. Thus, it can be concluded that the low oxygen concentration may result in selective pressure in favor of strains with a low oxygen dependency such as chalk yeasts [17].

### 3.3. Influence of Temperature and Aw on Chalk Yeast Growth on MEA

The influence of temperature and Aw on the growth of *W. anomala*, *S. fibuligera*, *H. burtonii*, and *C. fabianii* on MEA was investigated (Table 4).

In this study, the influence of pH was not considered as it represents a variable that does not change during the production and storage of GFB. The pH of both spoiled and unspoiled GFB was always about 5.34 ± 0.04 units. This value does not represent the optimum pH for the growth of all the isolated strains. For the investigated yeasts, different authors have shown that the optimum pH for their growth is between 4.5 and 4.8; however, their growth rate was significant at a pH value between 2.8 and 8.0 [17,52,55]. Moreover, our experience suggests that pH does not play a significant role in the control of the growth rates of yeasts, particularly of chalk yeasts, as demonstrated by several authors [11,17,23,56]. Only Arroyo et al. [56] reported that pH seems to influence the lag phase of *W. anomalus*.

The growth temperature did not seem to influence the investigated chalk yeasts (Table 4). No significant differences were observed regarding the yeast growth at the tested temperature (20–25 °C) and at different Aw levels (*p* > 0.05). Only *H. burtonii* demonstrated significant differences in its growth at the tested temperature (20–25 °C) and at the Aw levels of about 0.99, 0.98, and 0.97 (*p* < 0.05). The obtained results agreed with those of the studies of Debonne et al. [11]. However, it can be assumed that the investigated temperature was less than the temperature required for the optimal growth of chalk yeasts. According to different authors [57,58], the optimal growth temperature of *S. fibuligera* and *H. burtonii* is 30 °C. In addition, our data were confirmed by Debonne et al. [11] who observed only a slight difference in the maximal growth rate between 22 and 30 °C for three out of the four chalk yeasts that we studied. Furthermore, they showed that *H. burtonii* and *S. fibuligera* had very similar growth rates at 22 and 30 °C, and only the growth of *W. anomalus* was significantly high at 30 °C.

The Aw values influenced the growth of all the tested chalk yeasts. As expected, the decrease in the Aw produced a decrease in the strain growth (Table 4) at both temperatures (*p* < 0.05). Data demonstrated that a low Aw reduced but did not inhibit the growth of all the tested strains, confirming the results reported by various authors. Furthermore, *W. anomalus* (*P. anomala*) can also grow at an Aw of 0.88 after a long lag phase of about 11.5 days [17]. However, according to Fredlund et al. [59] and Lahlali et al. [60], its minimum Aw values were about 0.92 and 0.93, respectively. Consequently, it can be assumed that the minimum or maximum Aw growth depends on the strains. Indeed, Legan and Voysey [52] showed a minimum Aw value of 0.75 for *W. anomala* growth. The minimum Aw that allows growth seems to be strain-dependent. Our findings regarding *H. burtonii* confirmed the results of Simoncini et al. [61], who showed that it grew on dry-cured ham, having an Aw level of about 0.911. Again, *S. fibuligera* grew slightly at an Aw of about 0.90 (Table 4) as just observed by Deschuyffeleer et al. [17], who confirmed that the minimum Aw growth depended on the strain. Furthermore, they found that the isolated *S. fibuligera* strains grew at an Aw of 0.90, while the reference strains had a minimum Aw value for growth of 0.94, and they concluded that the isolated strain of *S. fibuligera* was more adapted to a lower Aw, thus exhibiting a higher spoilage potential. A colony with a diameter of 3 mm is sufficient to consider a product visually spoiled [62,63]. The spoiled GFB that was investigated presented colonies of different diameters, i.e., 2–3 mm to 3–10 cm. *C. fabianii* is the most sensitive strain in terms of Aw, reaching a diameter of 0.2 cm on MEA at 20 °C. Conversely, the other strains were less sensitive and showed good resistance towards the lowering of water activity. However, all the strains had lag phases of about 7 days at both temperatures before being visually observed (3 mm). Thus, our results about *H. burtonii* and *S. fibuligera* are in accordance with those of Deschuyffeleer et al. [17], who showed an inhibition for at least one week. Finally, the optimum Aw value was 0.99, but good growth was also observed at an Aw of 0.98, as just reported by Gibson et al. [62] and Lahlali et al. [60] for *H. burtonii* and *S. fibuligera* and by Deschuyffeleer et al. [17] for *W. anomala*. It can be assumed that the slight differences in the growth at various Aw levels could be partly explained by another strain that was used with another medium (MEA) in this study.

The behavior of *C. fabianii* at different temperatures and Aw was only partially discussed because, until now, it had never been isolated in GFB and was only isolated in GB for the first time last year [24].

### 3.4. Influence of Preservatives on Chalk Yeast Growth in MEA

Preservatives are most commonly used to control and prevent yeast and mold growth in baked goods [64,65]. Chemical preservatives, including acetic, sorbic, and propionic acids and their salts, have been proven to effectively inhibit bread spoilage by fungi [66]. However, recently, the use of chemical preservatives has been restricted in new resistant fungal strains due to their toxicity, poor solubility, and low potency [20]. Propionic acid and its salts such as calcium and potassium propionate are mainly used in bakery products to prevent yeast growth in bread and cakes. Table 5 shows the effects of natural preservatives (hop extract and ethanol) and synthetic preservatives (sorbic and propionic acids) in MEA. Sorbic acid is usually applied directly to bread dough since it does not have an action against yeast during the fermentation [67], while propionic acid and its salts are effective when used in industrialized bread, but some mold species show some resistance even at high concentrations of the preservative (0.3%) [20,21].

The concentrations of synthetic preservatives used in this study were selected based on literature data [68,69,70,71], i.e., the concentration of hop was obtained from Comi et al. [34] (0.08 mg CAE/mL product) and that of ethanol from Directive [25] (2% (*w*/*v*)), which is lower than the taste threshold limit of 1–2% (*w*/*v*) [72,73].

As observed, the preservatives did not completely inhibit the yeast growth. Each yeast strain showed different behavior according to the preservative used. In each case, the visibility of the colony appeared within one month. Propionic and sorbic acids could not be added at the maximum concentration level permitted by EU-directive 1129/2011/EC [74] but, as indicated above, a concentration of over 200 ppm modified the flavor of MBIC [75].

The lack of effectiveness of propionic and sorbic acids is probably due to the pH of the food matrix, which is 5.34. Thus, both acids are not sufficiently non-dissociated depending on the pKa [75]; consequently, they do not inhibit the yeasts. It is well known that the efficacy of organic acids and their salts is greatly influenced by the dissociation constant and by the pH of the substrate as they do not completely dissociate in water [67,76].

The ethanol and hop extract exhibited greater effectiveness compared to the synthetic preservatives; consequently, their effectiveness was further evaluated either in vitro or in vivo.

### 3.5. Vapor-Phase Antimicrobial Activity of Ethanol and Hop Extract

The method by Petchwattana et al. ([35], Modified) demonstrated that regardless of the concentration of both the preservatives and the temperature (20–25 °C), all the tested yeast strains could grow in MEA. Also, at the level of 2% (*v*/*w*), i.e., low concentration of both preservatives, it was not possible to observe any growth on MEA. Consequently, this concentration was used in vivo for both the preservatives.

### 3.6. Antimicrobial Activity of Hop Extract and Ethanol in Gluten-Free Bread

The GFB samples were divided into two groups. The hop extract solution (2% *v*/*w*) was added to the first group, and the ethanol solution (2% *v*/*w*) was added to the second group. Table 6 shows the time (in days) required to detect the stationary phase in days at 20 °C for the chalk yeast cocktails inoculated in gluten-free slice bread. As previously shown, no growth was observed in the GFB containing ethanol. Conversely, in the GFB containing hop extract, all the yeast strains demonstrated different times of the stationary phase, i.e., between 120 and 180 days. In particular, the time of the stationary phase was about 120 days for *S. fibuligera*, 140 days for *H. burtonii*, and 170 days for *C. fabianii* and *W. anomalus*. The yeasts grew in the control samples within 10–40 days.

Despite the presence of ethanol in hop extract, all the yeasts grew slowly. It can be assumed that hop extract represents a growth substrate for this kind of microorganism. Indeed, the antimicrobial effectiveness of hop extract was demonstrated only against bacteria. It is well known that hop bitter acids inhibit Gram-positive bacteria, including *Bacillus*, *Micrococcus*, *Staphylococcus*, and other bacteria [34,77,78,79]. Their inhibitory activity has also been reported for certain fungi [80].

The shelf-life of GFB is about 180 days. The experimental data demonstrated that the yeasts did not grow in the period up to 180 days, and the use of ethanol was suggested for preventing the growth of chalk yeasts.

### 3.7. Sensorial Analysis

Among the non-professional subjects, 8 out of 20 assessors recognized the different samples. This number is not statistically significant (*p* > 0.05). Indeed, for 20 assessors, the minimum number of correct judgments to establish significance at levels of *p* < 0.05 for the triangle test is 11 [40,41]. Despite the recognition of the different samples, all the tested samples were considered acceptable. On the other hand, the 12 assessors who did not guess the different samples considered all the tested samples acceptable.

Finally, the acceptability of ethanol-fortified breads confirms the suggestion to use this antimicrobial to prevent yeast growth.

### 3.8. Volatile Compound Analysis

Considering that all the yeast strains were present at the same time in the spoiled GFB, it was necessary to study volatile compounds to confirm their activity. Indeed, the levels of some alcohols, carboxylic acids, aldehydes, ketones, and esters increased in the spoiled GFB samples. This feature is emphasized in Table 7, which lists only the components whose concentrations varied considerably between the spoiled and the unspoiled GFB (*p* < 0.05).

Since the concentrations of hydrocarbons did not differ between the spoiled and unspoiled samples, they were not reported. Furthermore, the concentrations of 2 aldehydes, 4 alcohols, 3 esters, 3 acids, and 18 hydrocarbons did not change in either the spoiled or unspoiled GFB (*p* > 0.05). Conversely, the amount of ethanol in the spoiled samples was greater than that in the unspoiled GB samples, exhibiting a significant difference (*p* < 0.05). In addition, the concentrations of higher alcohols were different among the GB samples. In particular, their concentrations were greater in the spoiled samples (*p* < 0.05). Yeasts produce higher alcohols directly through sugar fermentation or amino acid degradation [81,82,83,84]. The higher alcohols detected in the spoiled GFB included 2-methyl-propanol, 3-methyl-butanol, and phenyl ethyl alcohol, which could originate from amino acids [84,85]. The acetic acid concentration in the spoiled GFB samples was quite similar to that in the unspoiled GFB samples, but it could be assumed that it was used by the yeasts to produce acetic esters [83]. Indeed, the concentrations of ethyl acetate, n-propyl acetate, 3-methyl butanol acetate, and propionic acid 2 methyl ethyl ester were greater in the spoiled samples (*p* < 0.05). Esters are formed via an intracellular process catalyzed by an acyl transferase or “ester synthase” [24,86,87]. The reaction requires energy provided by the thioester linkage of the acyl-CoA co-substrate. The most abundant acyl-CoA is acetyl-CoA, which can be formed either by the oxidative decarboxylation of pyruvate or by the direct activation of acetate with ATP [83,84,85,86,87]. The majority of acetyl-CoA is formed by the oxidative decarboxylation of pyruvate, while most of the other acyl-CoAs are generated by the acylation of free CoA catalyzed by acyl-CoA synthase (fatty acid metabolism) [83].

Finally, all the volatile compounds identified in the spoiled samples do not influence the healthiness of the bread, as they are present in many foods and fermented products such as wine [81,82,83,84,85]. Thus, the higher concentration of volatile compounds in the spoiled GFB compared to the unspoiled GFB only confirms the activity of yeasts. In this study, the compounds produced by yeasts confirm the data of Colautti et al. [24], who in spoiled gluten bread observed different volatile compounds such as higher alcohols (isobutyl alcohol, isoamyl alcohol, and 1-propanol), esters (ethyl acetate, n-propylacetate, ethyl butyrate, isoamyl acetate, and ethyl pentanoate), ethanol, and acetic acids produced by *C. fabianii*. It could be assumed that the differences in volatile compounds observed in both GB and GFB could be due to the different substrate compositions and the higher number of yeast strains in GFB (4 versus 1). The spoilage of bread is indicated by the presence of white spots and the increase in some volatile compound concentrations.

## 4. Conclusions

*H. burtonii*, *W. anomalus*, *S. fibuligera*, and *C. fabianii* were isolated from industrial gluten-free bread (GFB) purchased from a local supermarket. They are responsible for the spoilage of GFB and present as white powdery and filamentous colonies due to the fragmentation of their hyphae into short-length fragments (dust-type spots), a typical representation of spoilage produced by chalk yeasts. Among these yeasts, *C. fabianii* was isolated in GFB for the first time; it is a rare ascomycetous opportunistic yeast species with low virulence attributes, uncommonly implicated in bread spoilage. Temperature did not influence the in vitro yeast growth, while the growth was influenced by the Aw (from 0.99 to 0.90), which produced a reduction in growth. Different preservatives (ethanol, hop extract, and sorbic and propionic acids) reduced but did not inhibit the yeast growth. The vapor phase of the ethanol and hop extract completely inhibited the in vitro yeast growth at the tested temperature. However, only 2% (*v*/*w*) ethanol completely inhibited all the yeast strains, while the hop extract reduced their growth. In addition, its presence did not deeply change the sensorial characteristics of the bread as demonstrated by the twenty nonprofessional subjects who were not able to distinguish between GFB with or without ethanol added and considered both acceptable. The spoilage was also confirmed by the presence of various compounds including alcohols, ketones, organic acids, and esters, which were identified in higher concentrations in the spoiled samples than in the unspoiled samples. The concentration of acetic acid was lower only in the spoiled samples because this compound was consumed by yeasts to produce acetate esters, which are predominately present in the spoiled samples.

Finally, considering the poor results obtained using the typical antimicrobials permitted by law in bread production, if we exclude the use of ethanol, we can hypothesize the use of bioprotective microorganisms or their metabolites in future studies on GFB manufacturing. Bioprotection meets the consumer demand for more natural and/or peculiar food and is usually related to sourdough fermentation. The efficacy of LAB as antifungal agents should be studied, including their production of organic acids, promotion of competition for nutrients, and production of antagonistic compounds such as lactic, acetic, propionic, sorbic, and benzoic acids; hydrogen peroxide; diacetyl; ethanol; phenols; and other protein-based antimicrobial compounds such as bacteriocins like lactocin and nisin.

The currently available literature data on bioprotection in GFB are limited and conflicting; consequently, additional data should be collected on this topic.

## Figures and Tables

**Table 1:** Gluten-free inoculated samples treated with hop extract solution and ethanol.

Yeast Cocktails	Gluten-Free Bread	Hop Extract	Ethanol	Control
*Saccharomycopsis fibuligera*	9	3	3	3
*Hyphopichia burtonii*	9	3	3	3
*Wickerhamomyces anomalus*	9	3	3	3
*Cyberlindnera fabianii*	9	3	3	3

Note: The number indicates the number of inoculated bags; hop extract (2% *v*/*w*) and ethanol (2% *v*/*w*) were used.

**Table 2:** Physicochemical parameters of gluten-free bread.

Parameters	Value %
Moisture	45.0 ± 0.3
Fat	3.0 ± 0.5
of which saturated fats	0.5 ± 0.2
Carbohydrates	40.0 ± 1.7
of which sugar	1.6 ± 0.3
Protein	4.6 ± 0.3
Salt	1.1± 0.1
Ash	1.2 ± 0.1
Fiber	6.2 ± 1.2
pH	5.34 ± 0.04
Aw	0.978 ± 0.01
Kcal	205 ± 10/100 g product

**Table 3:** Chalk yeasts isolated from gluten-free spoiled bread.

Species	Accession Number	Number Isolates	%
*Saccharomycopsis fibuligera*	MK394133.1	60	40
*Hyphopichia burtonii*	MH532416.1	15	10
*Wickerhamomyces anomalus*	MN054504.1	24	16
*Cyberlindnera fabianii*	MN371966.1	51	34
Total isolates		150	100

**Table 4:** Growth of chalk yeasts at 20 and 25 °C up to one month on Malt Extract Agar.

Aw	*Wickerhamomyces anomalus*		*Cyberlindnera fabianii*		*Hyphopichia burtonii*		*Saccharomycopsis fibuligera*	
	20 °C	25 °C	20 °C	25 °C	20 °C	25 °C	20 °C	25 °C
0.99	3.1 ± 0.3 a	3.5 ± 0.1 a	2.1 ± 0.3 a	2.5 ± 0.1 a	3.5 ± 0.2 *a	3.9 ± 0.1 *a	4.7 ± 0.3 a	5.0 ± 0.3 a
0.98	2.3 ± 0.3 b	2.5 ± 0.1 b	1.3 ± 0.3 b	1.5 ± 0.1 b	2.3 ± 0.1 *b	2.8 ± 0.2 *b	4.2± 0.4 b	4.1 ± 0.2 b
0.96	1.8 ± 0.1 c	1.9 ± 0.1 c	0.8 ± 0.1 c	0.9 ± 0.1 c	1.8 ± 0.2 *c	2.3 ± 0.1 *c	2.3 ± 0.3 c	2.5 ± 0.2 c
0.94	1.3 ± 0.2 d	1.5 ± 0.1 d	0.5 ± 0.2 d	0.7 ± 0.1 d	1.4 ± 0.2 d	1.7 ± 0.3 d	1.9 ± 0.2 d	2.2 ± 0.1 d
0.92	1.1 ± 0.1 e	1.2 ± 0.3 e	0.3 ± 0.1 e	0.6 ± 0.3 d	1.2 ± 0.1 e	1.3 ± 0.2 e	1.5 ± 0.1 e	1.6 ± 0.2 e
0.90	0.5 ± 0.1 f	0.7 ± 0.1 f	0.2 ± 0.1 e	0.5 ± 0.1 d	0.5 ± 0.1 f	0.4 ± 0.1 f	0.5 ± 0.1 f	0.6 ± 0.1 f

Note: Colony diameter measured (cm) on MEA; data represent the means ± standard deviations of the total samples; mean * within each species line (following the values) is significantly different (*p* < 0.05); mean with the same letters within each column (following the values) is not significantly different (*p* < 0.05). Analyses were conducted in triplicate for each species and temperature.

**Table 5:** Time to detect visibility in days (1 cm diameter) at 20 °C for chalk yeast cocktails inoculated in Malt Extract Agar and stored for 1 month.

Strains	Preservatives				
	Hop Extract	Sorbic Acid	Propionic Acid	Ethanol	Control
*Saccharomycopsis fibuligera*	28 ± 2 a	25 ± 1 b	25 ± 1 b	29 ± 2 a	18 ± 1 c
*Hyphopichia burtonii*	26 ± 1 a	26 ± 2 a	27 ± 1 a	28 ± 3 a	15 ± 2 b
*Wickerhamomyces anomalus*	28 ± 3 a	21 ± 2 b	22 ± 2 b	28 ± 3 a	15 ± 2 c
*Cyberlindnera fabianii*	24 ± 2 a	19 ± 3 b	21 ± 1 b	25 ± 2 a	10 ± 2 c

Note: Hop extract: 2% *v*/*w*; sorbic acid: 2000 ppm; propionic acid: 2000 ppm; ethanol: 2% *v*/*w*; control: no preservative; data represent the means ± standard deviations of the total samples; means with the same letters within each line (following the values) are not significantly different (*p* < 0.05). The analyses were conducted in triplicate for each strain, preservative, and control.

**Table 6:** Time (days) to detect stationary phase in days at 20 °C for chalk yeast cocktails inoculated in gluten-free slice bread.

Strains	Preservatives		Control
	Hop Extract	Ethanol	
*Saccharomycopsis fibuligera*	120 ± 5 a	No growth	40 ± 3 a
*Hyphopichia burtonii*	140 ± 7 b	No growth	20 ± 2 b
*Cyberlindnera fabianii*	170 ± 9 c	No growth	10 ± 1 c
*Wickerhamomyces anomalus*	170 ± 4 c	No growth	20 ± 1 b

Note: Hop extract: 2% *v*/*w*; ethanol: 2% *v*/*w*; control: no preservative; no growth up to 180 days. Data represent the means ± standard deviations of the total samples; means with the same letters within each column (following the values) are not significantly different (*p* < 0.05). The analyses were conducted in triplicate for three different samples for each strain, preservative, and control.

**Table 7:** Volatile compounds in spoiled and unspoiled bread.

Volatile Compounds	Spoiled	Unspoiled
	Mean ± Std Dev	Mean ± Std Dev
**Hexanal**	0	1.83 ± 1.87
2-Heptenal	0	0.32 ± 0.36
Furfural	0	0.12 ± 0.12
Benzaldehyde	0.38 ± 0.12	1.09 ± 0.32
ALDEHYDES *	**0.38** ± **0.11**	**3.36** ± **2.67**
Acetoin	0.14 ± 0.12	0.63 ± 0.71
KETONES *	**0.14** ± **0.12**	**0.63** ± **0.71**
Ethanol	21.01± 2.16	6.51 ± 5.25
2-methyl propanol	1.13 ± 0.84	0.13 ± 0.22
3-methyl butanol	7.34 ± 1.99	2.77 ± 1.66
Phenyl ethyl alcohol	3.36 ± 1.21	2.39 ± 0.05
ALCOHOLS *	**32.84** ± **6.2**	**11.80** ± **7.18**
Ethyl acetate	5.62 ± 8.86	0.34 ± 0.58
Propanoic acid. 2-methyl. ethyl ester	0.22 ± 0.23	0
n-propyl acetate	0.15 ± 0.26	0
3-methyl butanol acetate	1.35 ± 1.53	0.5 ± 0.30
ESTERS *	**7.34** ± **10.88**	**0.84** ± **0.88**
Acetic acid	1.66 ± 1.64	1.44 ± 0.11
ACIDS *	**1.66** ± **1.64**	**1.44** ± **0.11**

Note: Data are expressed as the ratio between the area of each peak and the area of the internal standard (4-methyl, 2-pentanol); * sum of compounds; data represent the means ± standard deviations (Std Dev) of the total samples. All the compounds are significantly different in the spoiled and unspoiled GB (*p* < 0.05).

## Data Availability

The original contributions presented in the study are included in the article; further inquiries can be directed to the corresponding author.
